# The focus of attention in working memory—from metaphors to mechanisms

**DOI:** 10.3389/fnhum.2013.00673

**Published:** 2013-10-17

**Authors:** Klaus Oberauer

**Affiliations:** Department of Psychology, University of ZurichZurich, Switzerland

**Keywords:** working memory, attention, computational modeling

## Abstract

Many verbal theories describe working memory (WM) in terms of physical metaphors such as information flow or information containers. These metaphors are often useful but can also be misleading. This article contrasts the verbal version of the author's three-embedded-component theory with a computational implementation of the theory. The analysis focuses on phenomena that have been attributed to the focus of attention in WM. The verbal theory characterizes the focus of attention by a container metaphor, which gives rise to questions such as: how many items fit into the focus? The computational model explains the same phenomena mechanistically through a combination of strengthened bindings between items and their retrieval cues, and priming of these cues. The author applies the computational model to three findings that have been used to argue about how many items can be held in the focus of attention (Oberauer and Bialkova, 2009; Gilchrist and Cowan, 2011; Oberauer and Bialkova, 2011). The modeling results imply a new interpretation of those findings: The different patterns of results across those studies don't imply different capacity estimates for the focus of attention; they rather reflect to what extent retrieval from WM is parallel or serial.

## Introduction

There is broad agreement that representations in working memory (WM) are not all equal. Within a set of items or chunks in WM, a subset can be given privileged status, making them particularly easily and quickly accessible. Researchers have conceptualized this phenomenon by the notion of a focus of attention directed to a subset of the contents of WM. Theories of WM that assume a focus of attention differ in what functions they ascribe to the focus, and they also differ in the assumed scope of the focus. Whereas Cowan (1995), (2001), (2005) assumes that the focus of attention can hold up to about four independent chunks, McElree (2006; McElree and Dosher, 1989) has proposed a focus of attention limited to a single chunk.

In my own work I have proposed an integration of those two views into a framework that distinguishes three states of representations in WM: the activated part of LTM, the region of direct access, and the focus of attention (Oberauer, 2002, 2009). As in the theories of Cowan and of McElree, the activated part of LTM encompasses representations currently not in the central part of WM but easy to retrieve from LTM into central WM. The region of direct access roughly corresponds to the broad focus in Cowan's theory, with a scope of about four chunks. The direct-access region serves to represent a structure, that is, a set of elements (items) and their relations, established by temporary bindings. The direct-access region has a limited capacity that constrains the complexity of structure representations and thereby limits our reasoning ability (Oberauer et al., 2007). Different from the capacity limit of the focus of attention in Cowan's theory, the capacity limit of the direct-access region is not a fixed number of items or chunks that can be maintained, but rather arises from interference between temporary bindings. The focus of attention roughly corresponds to McElree's single-chunk focus. Different from McElree's focus, in my framework the focus of attention does not have a capacity limit that constrains it to a single item. Rather, the focus of attention is a selection device, the function of which is to select a single item or chunk from the set currently held in the direct-access region. The focus usually limits itself to a single item or chunk because taking in more would undermine its function (Oberauer and Hein, 2012).

So far, theories of representational states in WM have remained largely metaphorical, characterizing WM as a set of embedded containers that hold different subsets of information. This metaphorical theorizing has served the purpose of inspiring many fruitful research questions, but as we gain more detailed empirical knowledge about the causes and consequences of attending to contents of WM, we are beginning to notice the limitations of the container metaphor. I believe that it is time to move on to a mechanistic theory of WM, and of the role attention plays in WM. My colleagues and I have started to work on a computational implementation of the three-embedded-component framework as a connectionist model (Oberauer et al., 2013). One thing we learned during this work is that there is no simple mapping between the metaphorical talk of representations being “in” or “outside” the focus of attention and components or processes of the model. Representations have different states, and play different roles, at different points during cognitive work on a task, but the container metaphor does not capture these states and roles adequately.

In this article I will give a summary of our computational model, and use it to re-analyze data from three studies (Oberauer and Bialkova, 2009, 2011; Gilchrist and Cowan, 2011) that speak to an issue of theoretical debate on the focus of attention: can the focus hold more than one item at a time? Our previous interpretation of the findings of Oberauer and Bialkova (2009) was that the focus of attention in WM is limited to a single chunk. It can hold more than one item at the same time but only if those items are chunked. Other work suggested that two very different items (e.g., one digit and one spatial location) can be held in the focus simultaneously when they are bound into a single mental object (Bao et al., 2007; Oberauer and Bialkova, 2011). Gilchrist and Cowan (2011), using a paradigm similar to that of Oberauer and Bialkova (2009), presented data that they interpreted as evidence that the focus of attention can hold several not-chunked items at the same time. Thus, the results of these three studies imply different conclusions about how many items “fit into” the focus of attention. In light of our computational model the published interpretations of the three studies cited above require a revision. Both the question and the answers we and others proposed reflect the container metaphor, implying that an item is either in or outside of the focus of attention. We will see that in this instance the container metaphor obstructs a more complete understanding of the mechanisms of attention to WM contents.

### Three embedded components of working memory—a computational model

**Figure 1:**
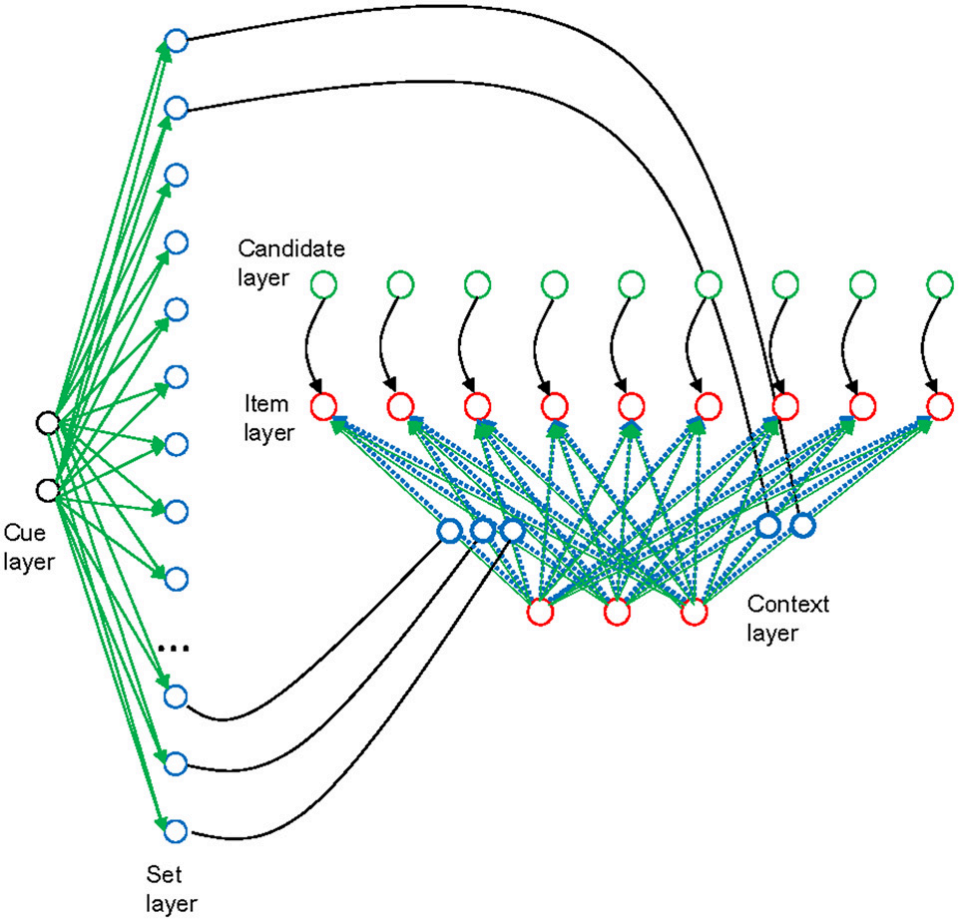
**Schematic outline of the model architecture [from Oberauer et al. (2013); reprinted with permission], applied to declarative working memory.** The item-selection module is depicted on the right side, with three layers of units arranged horizontally. Context layer and item layer are fully interconnected by rapidly updatable bindings (blue broken lines). In addition, they are also interconnected by slowly modifiable associations (green continuous lines), but these associations are not used in the present simulations. Each unit of the candidate layer has a fixed one-to-one connection to a corresponding unit in the candidate layer. The set-selection module is depicted on the left, with two layers of units arranged vertically. Each unit of the set layer is mapped to one binding in the item-selection module; a subset of these connections is shown (continuous black lines). The blue circles illustrate that the bindings might be neurally implemented as gain-modulating neurons, whose level of activation modulates the connectivity between two other neurons (Salinas and Thier, 2000). Thus, the strength of a binding is represented as the activation of a gain-modulating neuron, and it can be read out into, and in turn modified by, the corresponding unit in the set layer. The set layer is fully interconnected with the cue layer by slowly modifiable associations (green continuous lines), enabling learning of associations between memory sets and set cues. Green components in the figure reflect model components corresponding to the activated part of LTM, blue components correspond to the direct-access region, and red components correspond to the focus of attention in the three-embedded-components model (Oberauer, 2009).

We implemented the three-embedded-components model (Oberauer, 2009) as a connectionist model with two modules, an item-selection module and a set-selection module. The model architecture is shown in Figure 1. The architecture is intended as a model of both declarative WM and procedural WM. Declarative WM refers to WM for the objects of thought (such as symbols, physical objects, concepts), whereas procedural WM refers to WM for intended (cognitive or overt) actions on those objects (such as selecting a response to a stimulus, or moving a physical object in space). Here I apply the model to working-memory paradigms that require maintenance of memory sets in declarative WM, and cognitive operations on some of the items in declarative WM; these operations are controlled by task sets in procedural WM. Therefore, I will make use of two copies of the architecture in Figure 1, one for declarative and one for procedural WM. Here I first describe the architecture as a model of declarative WM.

The item-selection module in declarative WM implements the region of direct access and the focus of attention in the three-embedded components model. It serves to represent a single memory set, that is, a set of items bound to their specific contexts. An item can be any information unit known to the person, such as a letter, a word, or an object. A context is any information that can be used as a cue to selectively retrieve an item within a memory set, for instance the item's serial position in a list, its location in space, or its color. The item-selection module consists of three layers. The input layer represents the contexts that are used as retrieval cues, and I will therefore refer to it as the context layer. The output layer represents the items, and I will therefore refer to it as the item layer. In our applications of the model so far—including the ones presented here—we used localist representations of items and contexts, such that each item is represented by one unit of the item layer, and each context cue is represented by one unit of the context layer (though with some overlap to neighboring contexts). This does not reflect a theoretical assumption about the nature of representations but a decision made for modeling convenience.

Items are bound to contexts by a matrix of bindings, which links each unit of the item layer to each unit of the context layer. The binding strengths are rapidly modifiable by a variant of the Hebb learning rule called the delta rule. The delta rule updates the item-context bindings, simultaneously encoding new bindings and removing old bindings that are inconsistent with the new bindings. The third layer of the item-selection module is the candidate layer, which has fixed one-to-one connections to the item layer. Each unit of the item layer receives tonic activation from the corresponding unit of the candidate layer, thereby increasing its chances of being selected for retrieval. The activation level of each candidate unit reflects the degree to which an item is regarded as a candidate for retrieval.

Encoding a memory set involves activating each item in the item layer together with its context in the context layer, and encoding their relation by updating the binding matrix through delta-rule learning. Retrieval of an item starts with activating its context in the context layer. The context serves as a retrieval cue. Activation in the context layer is forwarded through the binding matrix to the item layer. At the same time each item unit receives input from the corresponding unit in the candidate layer. Each item unit gradually accumulates activation over time; this process is modeled by the linear ballistic accumulator (LBA) model (Brown and Heathcote, 2008). Thus, we can think of each item unit as an accumulator as described by LBA. The rate of activation accumulation in each item unit is governed by the drift rate, which is determined by the summed input to the item unit from the context layer and from the candidate layer. The first item unit whose activation reaches a boundary is selected for retrieval. Usually this is the correct item because it receives the largest input from the context layer. Because random noise is added to the drift rate of every unit in the item layer, the time until the boundary is reached varies from trial to trial [thereby generating variability in response times (RTs)], and occasionally the wrong item wins the race (thereby generating errors).

The set selection module serves to re-encode memory sets as chunks. After encoding a memory set, the binding matrix in the item selection module contains the information about that set as maintained in WM. The binding matrix can be read out into a vector of activation levels in the set layer of the set-selection module. Thus, the pattern of activation across the set layer codes the entire memory set as a single distributed representation. This layer is fully connected to a cue layer that represents cues for entire memory sets. Delta-rule learning in the set-selection module associates the activation pattern in the set layer to the currently activated set-cue representation in the cue layer. Thereby, the set-selection module enables the system to learn several memory sets. Each memory set can be retrieved by re-activating its set-cue in the cue layer, and thereby reproducing the activation pattern associated to it in the set layer. This activation pattern is fed back into the binding matrix of the item-selection module. The binding matrix is updated gradually by an iterative delta-rule learning process until the binding matrix matches the input pattern from the set-selection module. In this way, memory sets acquired as chunks can be retrieved and re-instated in the item-selection module.

We can now map the components of the connectionist architecture to the components of the three-embedded-components framework (Oberauer, 2009). The item currently active in the item layer, together with its context currently active in the context layer, can be thought of as the current content of the focus of attention. In the model there is no hard-wired constraint limiting the item layer and the context layer to holding only a single item or context representation at any time, but when multiple representations are being activated simultaneously in these layers, they risk being blended or confused with each other, which would be dysfunctional in most circumstances. Therefore, the content of the focus of attention is typically limited to a single item-context conjunction, but this limitation arises not from a capacity limit in the system, but from functional considerations: in many cognitive tasks, the function of the focus of attention is to selectively represent one item and one context, so that the item can be exclusively bound to its context at encoding, and the item can be exclusively selected as output at retrieval (Oberauer and Hein, 2012). We implemented this strong selectivity by two processing assumptions: Whenever encoding or retrieval of an item has come to completion, the activation in the item layer is entirely cleared. The activation in the context layer is squashed (i.e., multiplied by a value << 1) but not entirely cleared. As a consequence, contexts are represented less exclusively in the focus of attention than items—a point to which I return shortly.

The binding matrix corresponds to the region of direct access in the three-embedded-components model. The region of direct access is assumed to represent a small set of items by binding them to contexts (Oberauer, 2009). This function is accomplished by the binding matrix. The capacity of the direct-access region is limited by interference between item-context bindings. This interference is not yet fully implemented in the model—doing so requires distributed representations. My colleagues and I have modeled interference between item-context bindings in WM in a related model using distributed representations (Oberauer et al., 2012).

The activated part of LTM corresponds to several components of the connectionist model. First, the activation level of representations in the candidate layer reflects to what degree items are represented as potentially relevant for the current task, and are therefore primed so that they have a head start at retrieval. Second, the chunk representation of memory sets in the set-selection module reflects LTM for memory sets. More generally, the set-selection module enables long-term learning of representations of structures that have at one point been formed in the item-selection module, and that can be retrieved back into the item-selection module through appropriate set cues. The set-selection module enables the WM system to temporarily outsource some or all of its contents and bring it back later when needed (Oberauer, 2005; Lewis-Peacock et al., 2011; LaRocque et al., 2013).

### The object-switch cost and the focus of attention

One piece of evidence for the assumption of a single-item focus of attention in WM comes from the so-called *object-switch cost* (Garavan, 1998; Oberauer, 2003; Verhaeghen et al., 2004). When people are asked to carry out a sequence of cognitive operations, each of which requires access to an item in one particular context in WM, then they are faster when they need to access the same item-context conjunction as on the preceding step compared to when they need to switch to a new item in a new context. For brevity I will refer to an item-context conjunction in WM as an *object*. For instance, in one experiment (Oberauer, 2003) participants were asked to remember a set of four digits presented in four different colors, and then work through a series of arithmetic operations such as “+3” or “−5” to be applied to individual digits. Each operation was displayed in one of the four colors, which identified the item that the operation was to be applied to. RTs for arithmetic operations were faster when the operation had to be applied to the digit with the same color as the preceding operation than when it had to be applied to a digit with a different color. This effect—which can be described as an object switch cost or an object repetition benefit—has been explained by the assumption that the focus of attention selects the digit for the current operation, and after completion of the operation the item stays in the focus, so that it is immediately available for the next operation if that operation requires the same item as input, whereas it takes additional time for the focus to switch to another item.

**Figure 2:**
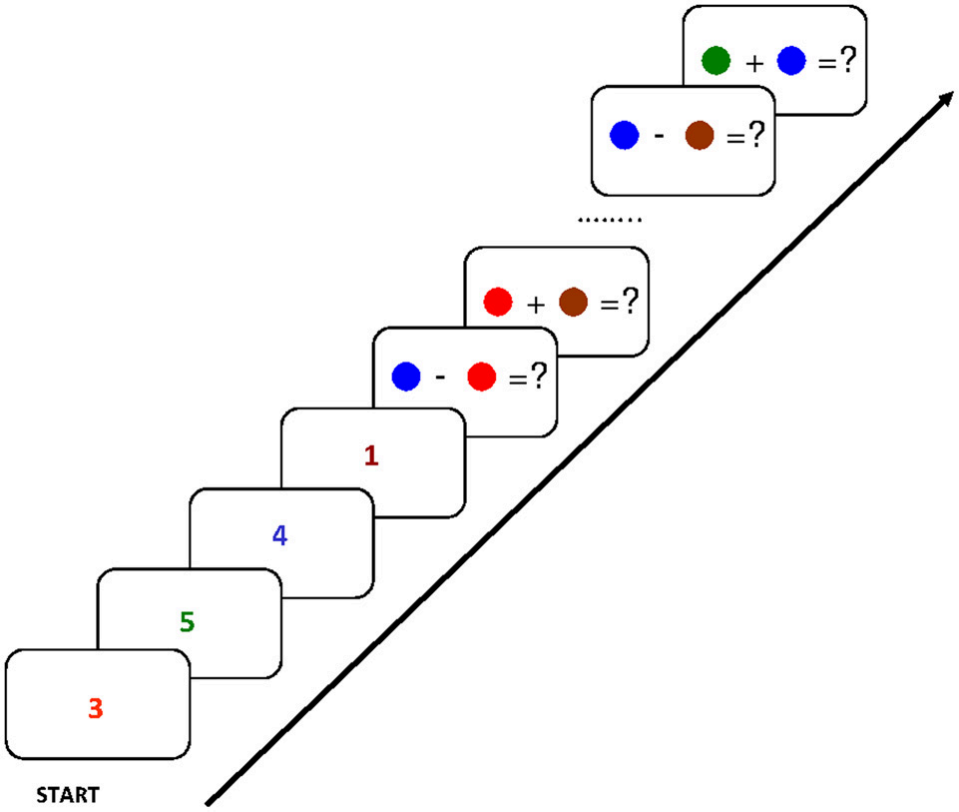
**Flow of events in the experiment of Oberauer and Bialkova (2009): After encoding of four digits in four different colors, equations were presented consisting of two colored dots, indicating the two digits from memory to be used, joined by a plus or minus sign.** Participants had to enter the result, upon which the next equation was presented. Figure reprinted with permission from Oberauer and Bialkova (2009).

Building on these findings and their explanation involving the focus of attention, Svetlana Bialkova and I asked what happens in the focus if access to two items in WM were required simultaneously (Oberauer and Bialkova, 2009). To study this situation we designed an experiment where participants again held four digits in mind, each associated to a different color (see Figure 2). We then asked them to work through a series of arithmetic problems in which both operands had to be retrieved from WM. The problems were displayed as two color patches, combined with an addition or a subtraction sign, such as [red] + [green]. Participants had to retrieve the red digit and the green digit and type the result of the addition as quickly as possible, upon which they were given the next problem. In this design, repetitions or switches could occur for the first or the second operand. For instance, starting from [red] + [green], presenting next the problem [red] − [yellow] is a repetition of the first operand, but a switch to a new digit for the second operand. Presenting next the problem [yellow] + [blue] is a switch to the other (i.e., the previous second) operand for the first operand, and a switch to a new digit for the second operand. The design fully crossed three kinds of transitions of the first operand (repeat, switch to other operand's digit, switch to new digit) with three kinds of transitions of the second digit (repeat, switch to other, switch to new). Out of the resulting nine transitions, two (repeat/switch-to-other, and switch-to-other/repeat) were eliminated because they resulted in problems using the same digit for both operands.

**Figure 3:**
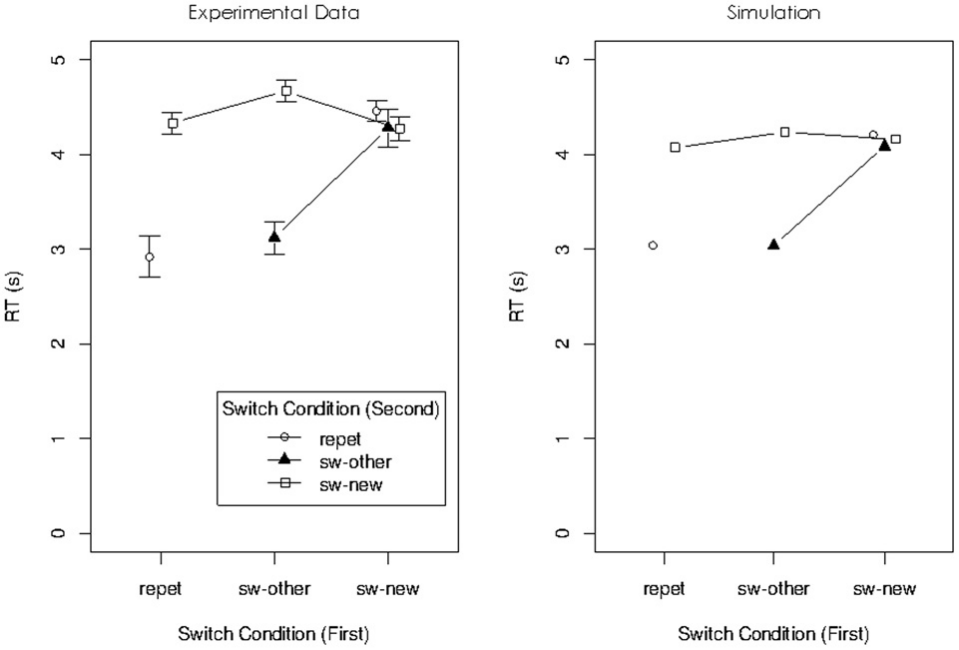
**Experimental data (left panel) and simulated data (right panel) of Experiment 1 in Oberauer and Bialkova (2009)**.

The mean RTs for the remaining seven conditions are presented in the left panel of Figure 3. There was a substantial repetition benefit in the two conditions in which both digits used in the preceding problem were used again, regardless of whether they were used again in the same operand roles (condition repeat/repeat) or in swapped roles (condition switch-to-other/switch-to-other). There was no benefit at all if only one of the digits from the preceding problem was used again. We interpreted this pattern as showing that the focus of attention can hold two digits at the same time, but only when they are chunked, thereby forming a single unit in WM. The transition conditions in which both digits repeated afforded re-use of the chunk, but in conditions in which only one digit repeated the chunk formed in the focus during the preceding operation cannot be re-used, so no repetition benefit was obtained. This interpretation makes sense within the container metaphor, but it has two limitations. First, it rests on an assumption that I find less than satisfying: The WM system must be assumed to chunk pairs of digits on the fly, within just a few seconds. If arbitrary pairs of items in WM can be chunked so rapidly, we need to ask why WM capacity cannot be expanded indefinitely by chunking items, thereby reducing the number of chunks to be held in WM. In fact, work by Cowan and colleagues has shown that chunking arbitrary pairs of items takes learning over much longer time intervals before the resulting chunks are treated as units in WM (Chen and Cowan, 2005; Cowan et al., 2012). Second, as I will explain next, our work with the connectionist model sketched above resulted in a new explanation for the object-switch cost, which is not easily applied to the dual-access paradigm of Oberauer and Bialkova (2009).

### A computational explanation of object-switch effects in working memory

One aim of our work with the connectionist implementation of the three-embedded-component model (Oberauer et al., 2013) was to explain the effects of repeated access to the same item in declarative WM. We argued that these effects are analogous to the effects of repeatedly selecting the same response in procedural WM. Repeating a response in a sequence of easy choice tasks is beneficial as long as the task remains the same, but there is a response-repetition cost when people switch to another task (Rogers and Monsell, 1995). Based on the assumption that declarative and procedural WM operate by analogous principles, we predicted that there should be an item-repetition benefit as long as the memory set remained the same, but an item-repetition cost if people switch to another memory set. For instance, if people retrieve the digit “3” from the memory set [2 5 3], and on the next trial again retrieve “3” from the same set, they are faster than when they retrieve another digit from that set. This is the well-documented object-switch cost, or object-repetition benefit (Garavan, 1998; Oberauer, 2003). However, if people retrieve “3” from set [2 5 3] and then switch to another memory set [3 6 8], and retrieve “3” from that set, we predicted them to be slower than when they retrieved another digit from the new set. This is what we found (Oberauer et al., 2013).

The finding that, under some circumstances, there is an item-repetition cost rather than an item-repetition benefit in WM challenges the idea that an item retrieved from WM remains in the focus of attention, so that it can be re-used immediately in the next processing step. We therefore developed a new explanation for object-repetition effects, which we implemented in the connectionist model: Retrieving an item (e.g., as input for an arithmetic operation) starts with activating that item's context in the context layer. In the paradigm of Oberauer (2003) and Oberauer and Bialkova (2009), where digits are cued by their color, the contexts are colors. The activated context serves as input into the binding matrix, returning a pattern of activation in the item layer, which drives the accumulation process that eventually leads to selection of an item for retrieval. After completion of the cognitive operation that used the retrieved item (e.g., after the response to the arithmetic operation has been entered), the item layer is cleared (i.e., reset to zero), so that no trace of the item representation remains in the focus of attention. Moreover, the selected item's activation in the candidate layer is actually reduced, so that retrieval of the same item in the next processing step is inhibited. This process implements response suppression, a common mechanism for avoiding perseveration in models of sequential behavior (Farrell and Lewandowsky, 2012). The representation in the context layer is not entirely cleared, however, but only squashed. In addition, every time an item is being retrieved, the binding between that item and the currently active context is strengthened by delta-rule learning.

To summarize, what carries over from one processing step to the next is not a representation of the item—to the contrary, the item representation is being suppressed. What carries over is a stronger binding between the retrieved item and its context, and residual activation of that context. When on the next step the same item is retrieved from the same memory set, then the same context is used as a cue, and the item is retrieved through the same item-context bindings. An item-repetition benefit occurs because the combined beneficial effects of cue priming and of strengthened bindings are stronger than the suppressing effect of item inhibition in the candidate layer. However, if the memory set is switched from one step to the next, and the same item is being retrieved from the new memory set, then there is no beneficial effect from cue priming (because the item has a different context in the new memory set), and no beneficial effect of strengthened bindings (because the new memory set is represented by an entirely different set of bindings). The only remaining effect is the inhibition of the previously retrieved item in the candidate layer. Therefore, repeatedly retrieving the same item after a memory-set switch produces an item-repetition cost.

This new explanation of the item-repetition benefit, and its reversal into an item-repetition cost in conjunction with memory-set switches, does not fit well with the container metaphor. The speed of access to an item in WM is not determined by whether it is “in” or “outside of” the focus of attention, but by the combined effects of activation of context representations, item representations (in the candidate layer), and strength of bindings. The new explanation works well for experiments in which a single item needs to be retrieved from WM at each step. But how can it be applied to the dual-access paradigm of Oberauer and Bialkova (2009)? This is the problem I address next.

Application of the model to the dual-access paradigm is not straightforward because it involves adding assumptions. Therefore, the following modeling work is not a strict test of the model in the sense of running the model to derive novel predictions that are tested against data. Rather, I explored various options to discover a parsimonious and plausible set of additional assumptions that enable the model to accommodate data that, at first blush, appear to challenge the model's core assumptions. As such, this work is a test of the model in a broader sense: It investigates not whether the model in its published form predicts the data from the dual-access paradigm, but rather whether it is compatible with these data.

## Modeling access to two representations in working memory

### Object-repetition effects in the arithmetic dual-access paradigm

**Figure 4:**
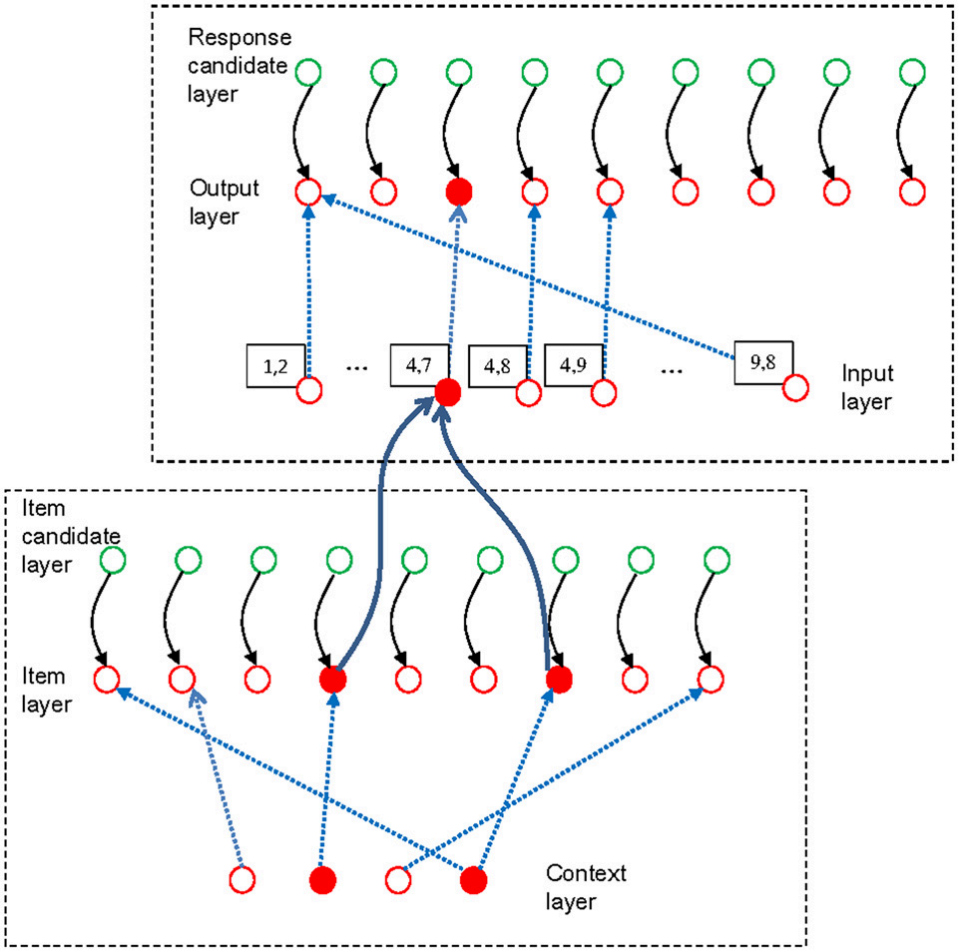
**Schematic illustration of the model applied to the arithmetic dual-access paradigm.** The bottom-left frame encloses the item-selection module of declarative working memory, and the upper-right frame contains the response-selection module of procedural working memory. The figure shows the state of the model at the end of one trial, in which the digits 4 and 7 have been retrieved from a memory set that includes the digits 1, 2, 4, and 7, each bound to a color context in the context layer. In deviation from Figure 1, the blue broken lines here represent only the bindings strengthened by encoding the list. The two retrieved items (filled red circles in the item layer) have activated the chunk [4, 7] in the input layer of the response-selection module (Only a subset of the chunk units in the input layer is shown). The binding matrix of the response-selection module has been configured for the subtraction task, thereby mapping the [4, 7] chunk to the result unit representing 3. Therefore, the output unit 3 is activated.

The existing experiments with the dual-access paradigm do not involve switching between multiple memory sets. Therefore, we need only the item-selection module of the connectionist model to model retrieval of items from WM in this paradigm. To account for the effect of chunking, however, we need to model more completely than before the processes involved in each trial of the dual-access paradigm as used by Oberauer and Bialkova (2009). We distinguish two processing steps: The first is to retrieve the two digits bound to the two colors given in the current arithmetic problem. This step is carried out in the item-selection module of declarative WM. The second step is to calculate the sum or the difference of the two retrieved digits, and produce the result as response. This step is carried out in the response-selection module of procedural WM. The response-selection module is the analogous counterpart of the item-selection module in procedural WM. Figure 4 presents a sketch of the model architecture relevant for the simulation of the arithmetic dual-access paradigm.

The architecture of the item-selection module is the same as in Oberauer et al. (2013). Its context layer consists of representations of the four colors that serve as retrieval cues for the digits. The colors are represented in semi-localist fashion: Each color is a vector of activation values across the context layer that peaks at one unit, unique for that color, and extends to neighboring units with an exponentially declining gradient. In this way, color representations overlap to some extent, reflecting their similarity. The item layer represents the nine digits in a localist fashion: Each digit is represented by one unique active unit, with all other units' activation set to zero. The two layers are interconnected by the declarative binding matrix, which is updated through the delta rule.

In each trial, the item-selection module retrieves two digits. These two digits are passed on to the response-selection module. The response-selection module implements the arithmetic task required in a given trial, that is, to compute the sum or the difference between two digits. Hence, it needs to map pairs of digits to either their sum or their difference. The input layer of the response-selection module consists of chunk units representing pairs of digits, and the output layer consists of units representing individual digits. I assume that the chunk units are the product of the person's learning history with addition and subtraction problems^1^. A second outcome of this learning history is the acquisition of task sets for addition and subtraction. Both these task sets consist of sets of bindings between digit-pair chunks and the corresponding results (i.e., their sum or their difference). On each trial, the operation sign in the equation (plus or minus) is used as a task cue that serves to retrieve the appropriate task set from the set-selection module of procedural WM, and implements it as a set of bindings in the binding matrix of the response-selection module. This process of task-set retrieval and implementation is of no concern in the present context and is therefore not explicitly modeled in the present simulations (see Oberauer et al., 2013, for a simulation of task switching).

The two digits retrieved in the item-selection module jointly activate the chunk unit in the response-selection module that represents that pair of digits. Each digit unit in the item layer of the item-selection module is connected to each chunk unit representing a pair of which that digit is a member. To ensure that only chunk units representing both retrieved digits as a pair are highly activated, the chunk units have an activation threshold that is surpassed only by the sum of the input from two digit units. I modeled the threshold by a logistic function that reflects some degree of noise in the threshold, or the input, or both, such that chunk units receiving input from both retrieved digits become strongly activated, and chunk units receiving input from only one digit are still activated, but to a much lesser degree. In this way, on the majority of trials the activated chunk units produce the correct response in the output layer of the response-selection module, but occasionally calculation errors can occur that tend to consist of results to an arithmetic problem that differs in one operand from the actual problem (e.g., responding to 3 + 6 or 2 + 5 instead of 3 + 5).

**Figure 5:**
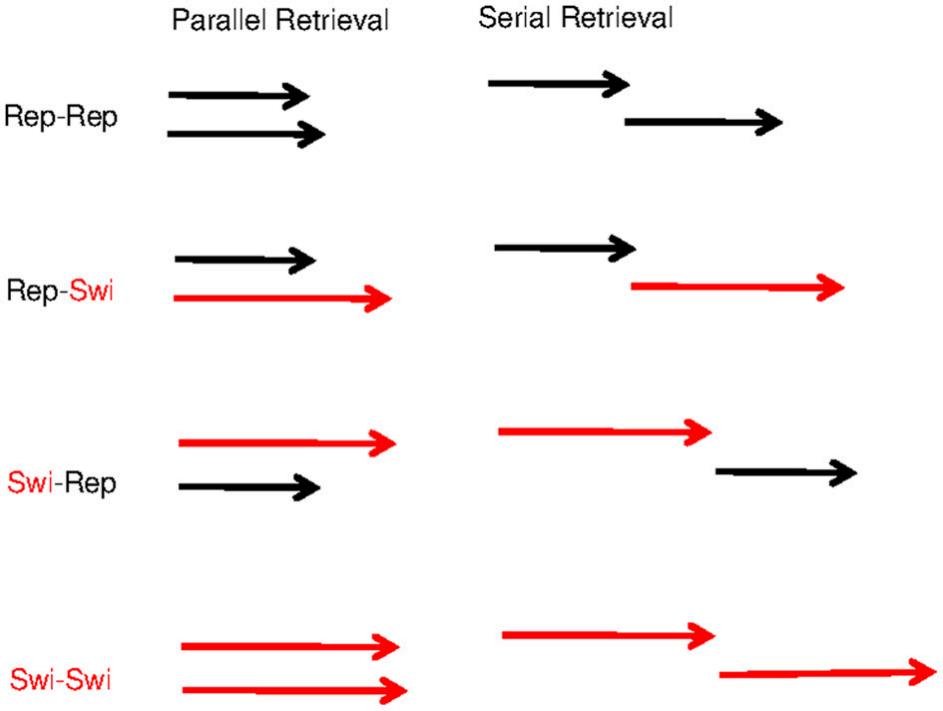
**Schematic illustration of the item-repetition effects on the assumption of parallel retrieval (left) and on the assumption of serial retrieval (right).** Each pair of arrows shows the duration of retrieving a pair of items. Black arrows represent retrieval of repeated items, and red arrows represent retrieval of not-repeated (i.e., switched) items, which on average takes longer. Response times are represented by the horizontal distance from the left-most arrow base (beginning of the trial) to the right-most arrow head (completion of both retrievals). The switch cost is additive with serial retrieval, but under-additive (i.e., the repetition benefit is over-additive) with parallel retrieval.

When modeling retrieval of two items from the direct-access region of WM, we need to consider whether they are retrieved serially or in parallel. It turns out that, at least within the present model architecture, the over-additive pattern of repetition benefits observed by Oberauer and Bialkova (2009) can only be explained by assuming parallel retrieval. The reason for this is straightforward: If we assume that the two digits are retrieved serially, then the completion time is the sum of the times for retrieving each digit. Whenever one digit is repeated from the preceding trial, retrieval of that digit is accelerated by priming of its retrieval cue (i.e., the color, which is necessarily also repeated) and by the strengthened binding between that digit and its color. When both digits are repeated, retrieval of both digits becomes faster. Importantly, the beneficial effects of digit repetition on RT combine additively when retrieval is serial. In other words, serial retrieval implies additive repetition benefits, contrary to what we observed (Oberauer and Bialkova, 2009). In contrast, when retrieval is parallel, the completion time is the maximum of the retrieval times of both digits. If one digit is repeated from the preceding trial but the other is not, then retrieval of one digit is accelerated, but this translates into only a minor benefit for the completion time because digit retrieval is complete only when both digits have been retrieved. Completion time is determined by the slower of the two retrieval times. Therefore, there is little repetition benefit when only one of the two digits is repeated, whereas there is a much larger repetition benefit when both are repeated. Figure 5 illustrates the consequences of serial and parallel retrieval for the predicted pattern of item-repetition benefits. This analysis is not specific to the present computational model—it applies to all models that assume a repetition benefit for retrieval times of individual items (Townsend and Nozawa, 1995).

For these reasons I assume that retrieval of the two digits in the arithmetic dual-access paradigm occurs in parallel. Item retrieval finishes when two digits have been retrieved, that is, when the accumulators of two units have reached the boundary. At that point in time, activation is passed from the item-selection module to the chunk units of the response-selection module. Response selection is a single process of selecting one digit as the result of the arithmetic operation. Hence, response selection finishes when the first unit in the output layer of the response-selection module reaches the boundary. The simulated response time is the sum of the time for retrieving the two items and the time for selecting the response.

Digit-repetition benefits arise from priming of retrieval cues and strengthening of bindings in both declarative and procedural WM. The retrieval cues are the representations in the input layers of the item-selection module (i.e., the colors) and in the response-selection module (i.e., the chunks of digit pairs). In declarative WM, the colors used in trial *n*-1 remain primed in trial *n*, and the bindings between the digits retrieved in trial *n*-1 and their colors remain strengthened. In procedural WM, a residual amount of the chunk unit activation from trial *n*-1 carries over as chunk priming into trial *n*. In each trial the binding between the selected response and the chunk unit active in procedural WM is also strengthened. This, however, has no effect in the present simulations because we analyzed only trials in which the arithmetic operation switched from the preceding trial, and operation switches imply a reconfiguration of the arithmetic task set, which undoes the strengthening of bindings from the preceding trial.

To summarize, in trials in which both digits are repeated from the preceding trial (though with a different operation), a repetition benefit arises from three sources: Retrieval of both items is accelerated by color priming and by strengthening of the digit-color bindings. In addition, chunk priming accelerates response selection. These three effects together explain the large RT benefit in the two conditions where both digits are repeated. It does not matter whether the digits are repeated in the same order or in the reverse order in the equation because digits are retrieved in parallel, so that color priming and strengthening of bindings affect digit retrieval regardless of their order. Chunk priming also benefits response selection when the digits are repeated in reversed order because chunks represent pairs of digits without order. This is possible because the sum and the absolute difference of two digits are independent of their order^2^.

In trials in which a single digit is repeated from the preceding trial, retrieval of that digit is accelerated by color priming and strengthened color-digit binding. Because item retrieval has to wait for the slower of the two digits to be retrieved, that benefit translates into only a small RT benefit^3^. This benefit is counteracted by the inhibition of the repeated digit in the candidate layer. Moreover, in single-repetition trials there is no benefit of chunk priming for response selection. The net effect of repeating a single digit is virtually zero.

The right panel of Figure 3 shows simulated data for Experiment 1 of Oberauer and Bialkova (2009). I used the parameter values from our previous simulations (Oberauer et al., 2013) except for three changes: Because the item-repetition effects in the dual-access paradigm are comparatively large, I increased the effect of cue priming by raising the proportion of cue activation carrying over into the next trial from 0.12 to 0.17, and I increased the effect of binding strengthening by raising the rate parameter of delta learning from 0.3 to 0.5. Moreover, because RTs in the experiments simulated here were much slower than those in Oberauer et al. (2013), I increased the boundary for the evidence accumulation process from 1 to 2 for all simulations in this article. Figure 3 shows that the model accurately reproduces the data pattern.

### Serial and parallel access to items in working memory

The success of the model in explaining the data from the arithmetic dual-access paradigm hinges on the assumption that in this task two digits are retrieved from WM in parallel. This assumption does not necessarily hold under all circumstances. We next apply the model to a paradigm in which participants are asked to access two items from WM and carry out two separate operations on them (Oberauer and Bialkova, 2011). Participants in this experiment initially encoded two digits, one associated to a piano tone, and the other to a trumpet tone, and in addition they encoded the locations of two dots in a matrix, one red and one blue dot. Subsequently they carried out a series of updating steps on items in WM. Arithmetic updating operations were given by a high tone (meaning “add two”) or a low tone (“subtract one”) played by either a piano or a trumpet. The musical instrument served as the cue to identify the digit to be updated. Spatial updating operations were given by centrally presented red or blue arrows pointing in the direction of the required shift; for instance a left-pointing arrow instructed the participant to shift the dot to the left by one matrix cell. The arrow color served as the cue to the to-be-updated dot. In the single-operation conditions of the experiment, each updating step involved either an arithmetic operation on one digit, or a mental shift of one dot in the matrix. In the dual-operation condition, which is of primary interest here, one arithmetic operation (i.e., a high or low trumpet or piano sound) and one spatial operation (i.e., a red or blue arrow) were presented simultaneously. Participants had to carry out both updating operations before they pressed the space bar once, upon which the next pair of updating operations was presented. Comparison of RTs in the dual-operation condition to those in the single-operation conditions revealed that, even after 36 sessions of practice, people could not carry out two updating operations in parallel without slowing relative to the single-operation condition. This implies that people either carried out the two operations serially, as predicted by a bottleneck model (Pashler, 1994), or that they carried out the updating operations in parallel but at a substantially reduced rate, as predicted by resource-sharing models (Navon and Miller, 2002; Tombu and Jolicoeur, 2003). This result contrasts with a previous study in which people practiced updating a single dot and a single digit, and achieved perfect time-sharing after practice (Oberauer and Kliegl, 2004).

**Figure 6:**
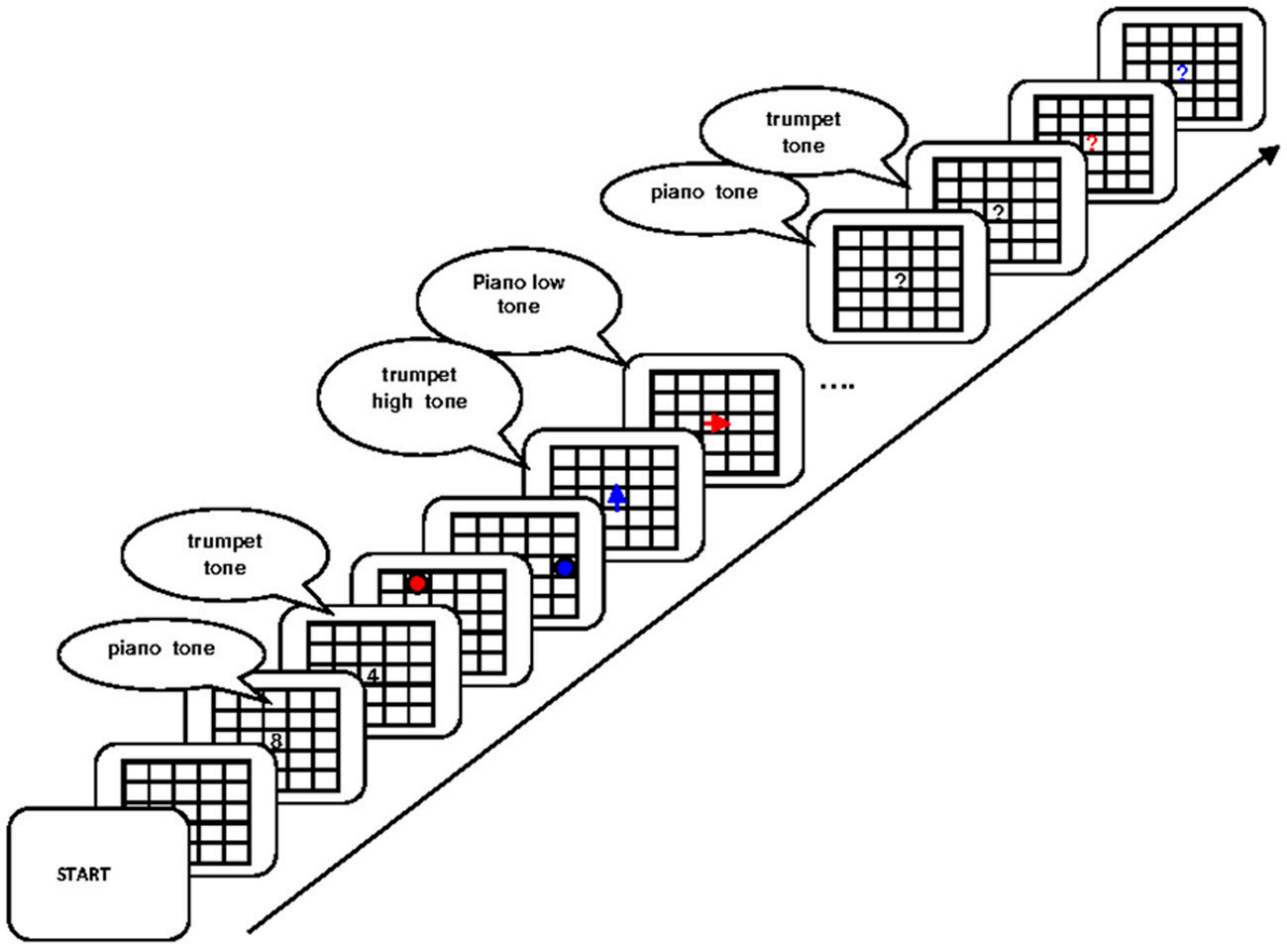
**Flow of events in the experiment of Oberauer and Bialkova (2011).** Initially participants encoded two digits, one associated to a piano tone and the other to a trumpet tone, and they encoded two dot positions in a matrix, one red dot and one blue dot. They then worked through a series of updating steps. Each step consisted of one tone (piano or trumpet), indicating the digit to be updated, and played in high or low pitch, indicating the updating operation (+2 or −1). Simultaneously, an arrow was presented in red or blue, the color indicating the to-be-updated dot position. Participants had to mentally update the digit and the dot position and press the space bar when ready, upon which the next tone and the next arrow are presented simultaneously. Figure reprinted with permission from Oberauer and Bialkova (2011).

**Figure 7:**
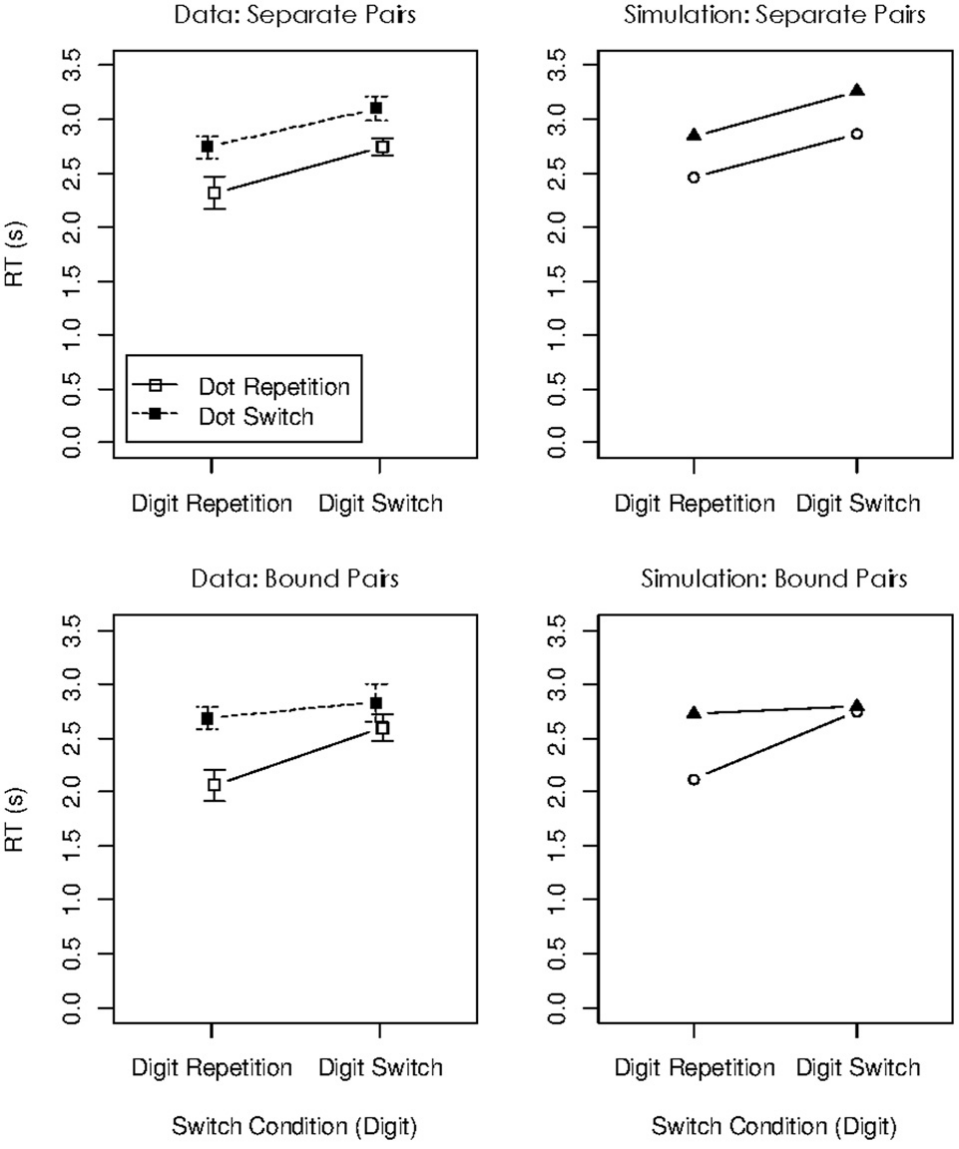
**Experimental data (left) and simulated data (right) for the dual-operation condition of Oberauer and Bialkova (2011)**.

The dual-operation condition of Oberauer and Bialkova (2011) is similar to the dual-access paradigm in that it requires access to two items in WM on every step (see Figure 6). We can therefore again look at object repetition effects in four conditions: Updating steps in which both digit and dot repeat from the preceding step, trials in which only the digit repeats, trials in which only the dot repeats, and trials in which both digit and dot are switched. This analysis revealed two different patterns, depending on which two items were updated. Previous research (Bao et al., 2007) as well as informal post-experimental interviews suggested that participants mentally bound the first-presented digit with the first-presented dot, and the second-presented digit with the second-presented dot. On updating steps involving two not-bound items, repetition effects were additive: Repeating either the digit or the dot yielded a modest benefit, and repeating both yielded an RT benefit twice as large (Figure 7, top). In contrast, when two bound objects had to be updated, the object-repetition effects showed an over-additive pattern mirroring that in the arithmetic dual-access paradigm (Oberauer and Bialkova, 2009): A repetition benefit was observed only when both digit and dot repeated from the preceding updating step (Figure 7, bottom). As discussed in the context of the dual-access paradigm, additive item-repetition benefits imply serial access to the two items in WM, whereas over-additive repetition benefits imply parallel access. We can conclude that in the dual-operation updating paradigm people access objects in WM serially, unless the two items, or their two cues, are bound together. It is not clear what it means to “bind” two items, or two cues, but it appears to be different from the acquisition of chunks because it can occur on the fly without much practice (Bao et al., 2007).

**Figure 8:**
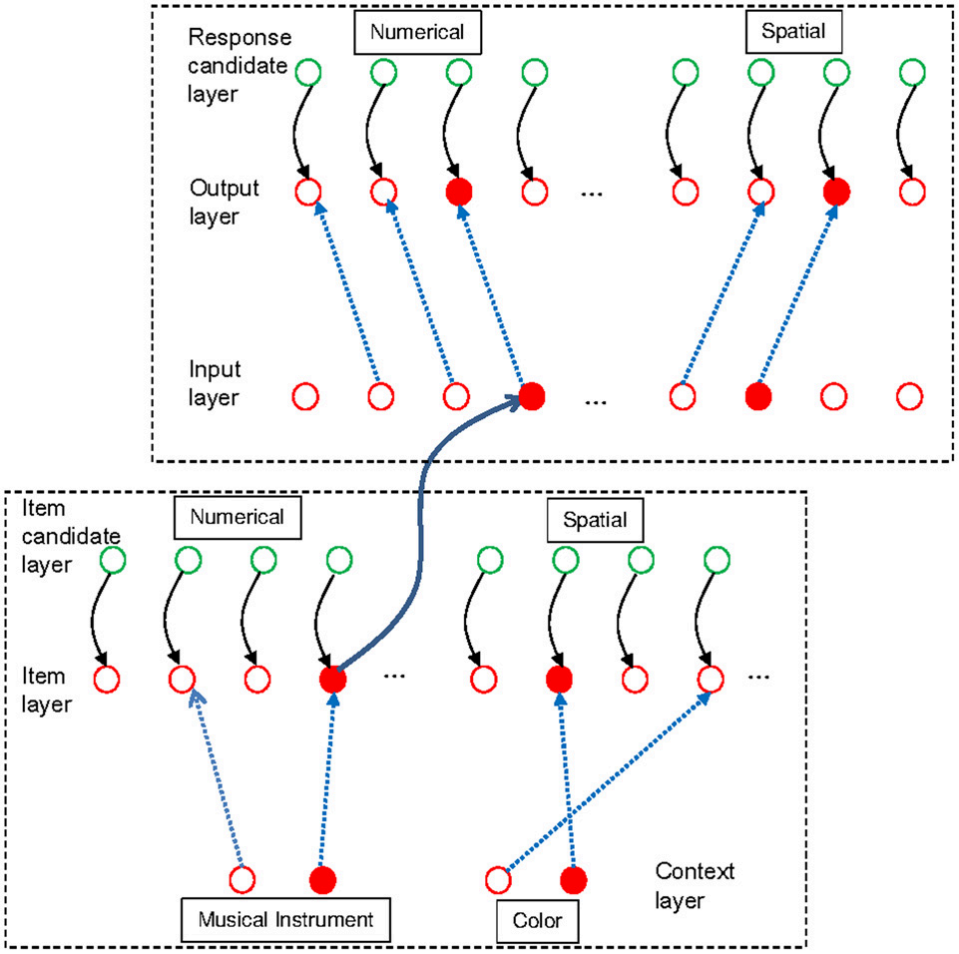
**Model architecture applied to the dual-operation condition of the experiment of Oberauer and Bialkova (2011).** As in Figure 2, the bottom-left frame surrounds the item-selection module of declarative working memory, and the upper-right frame encloses the response-selection module of procedural working memory. Each layer in both modules is separated into two sub-layers: The context layer consists of two units representing musical instruments, and two units representing colors. The musical instruments are connected to nine item units representing digits (only four of which are shown), and the color units are connected to nine item units representing dot locations (only four shown). The input and output layers of the response-selection module are analogous to the item layer. The figure shows the state of the model after completion of one updating step. One musical instrument and one color have been activated in the context layer. These activations have been forwarded to the item layer through the declarative binding matrix, which binds one digit to each musical instrument, and one location to each color. The retrieved digit activates the corresponding digit, and the retrieved location activates the corresponding location, in the input layer of the response-selection module. The procedural binding matrix is configured to carry out the required arithmetic updating operation (subtract one) in the numerical sub-module, and the required spatial updating operation (shift to the right) in the spatial sub-module. The activation in the input layer is forwarded through these bindings to the output layer, resulting in the selection of a new digit and a new location.

The model for the dual-operation condition of the updating task shares many architectural features with the model of the dual-access paradigm (see Figure 8). The context layer has four units, two for the two musical instruments that serve as cues for the digits, and two for the two colors that serve as cues for the dots. The item layer consists of nine units for the nine possible digits values, plus nine units for the nine possible dot locations in the matrix. Each item-layer unit is connected to a corresponding unit in the candidate layer. After encoding of the initial two digits and dot locations, each musical-instrument unit is bound to one digit, and each color unit is bound to one location.

An updating step begins with activating the unit for the musical instrument played in this step, and the unit for the color of the arrow displayed in this step. The degree of activation of these two units is determined by a vector of cue weights, with one weight for the musical instrument and one for the color. These cue weights act as filters between the perceptual input and activation of cue representations in the context layer; they can be interpreted as the degree to which the person pays attention to the tone characteristics and to the arrow color, respectively. The cue weights control the degree to which items are retrieved in parallel. With cue weights [1, 0], only the musical-instrument units are initially activated, implying that initially only the digit is retrieved. Once the digit is retrieved (i.e., once the first of the nine digit accumulators reaches the boundary), the cue weights are temporarily reversed to 1 minus the previous cue weights, resulting in [0, 1], and activation filtered by the new cue-weight vector is added to the context layer. As a result the color unit representing the arrow color is now also fully activated in the context layer (while the musical-instrument unit remains activated). Retrieval continues until the first location accumulator reaches the boundary. In this scenario, retrieval is completely serial, starting with the digit, followed by the location. The reverse order is of course equally possible, and in the simulations the order of the initial cue weight vector is determined at random for each updating step, with equal probability for both orders.

In contrast to the serial retrieval settings described above, the cue weight vector [1, 1] implies perfectly parallel retrieval. This setting is identical to the one implemented in the model of the dual-access paradigm—in that model I did not mention the cue weights because they were implicitly fixed to [1, 1]. With this setting, both the musical-instrument and the color units in the context layer are fully activated by the perceptual input from the start. Retrieval proceeds until the evidence accumulation in the first digit unit and the first location unit in the item layer has reached the boundary, implying that one digit and one location has been retrieved.

In between the two extreme cases described above there is a continuum of cue-weight settings that imply semi-parallel retrieval. For instance, cue weights [1, 0.4] mean that initially the musical-instrument units are fully activated by the heard tone, whereas activation of the color units is dampened to 40% of their maximum. With these settings, most likely a digit will be retrieved first, but it could happen by chance that a location is retrieved first. As soon as the first accumulator in the item layer (be it a digit or a location unit) reaches the boundary, the cue weights are reversed to [0, 0.6] so that now the appropriate color unit in the context layer is activated to its maximum (the musical-instrument unit is still fully activated). Again, retrieval finishes when the first digit and the first location have been retrieved.

In the simulations of the dual-operation condition of Oberauer and Bialkova (2011), the initial cue weights for each updating step are adjusted from one step to the next. When the musical instrument and the color belong to a bound pair, the initial cue weights are set to [1, 1], enabling fully parallel retrieval. When the musical instrument and the color belong to a separate (i.e., not bound) pair, the initial cue weights (before reversal) from the preceding step are adjusted toward more serial retrieval: The weaker of the two cue weights (or in case of equal weights, one selected at random) is reduced according to

(1) $$C_{w}(n)=C_{\text{min}}+{\left(1-C_{\text{min}}\right)}^{*}C_{w}{\left(n-1\right)}^{*}C_{r}$$

where *C*_w_ is the to-be-adjusted cue weight, *n* is the trial number, *C*_min_ is the minimum cue weight, and *C_r_* is the proportional cue-weight reduction factor. This adjustment rule involves two new free parameters, *C*_min_ = 0.4, and *C*_r_ = 0.8. By this adjustment rule, retrieval becomes more and more serial across subsequent updating steps involving separate pairs of cues, but whenever a bound pair is presented, it instantly returns to completely parallel retrieval. One way in which the return to parallel retrieval could occur is by having a strong association between cue weights for bound units in the context layer, such that the two cue weights boost each other up to their maximum level of 1.

As soon as one item (either digit or location) has been retrieved, it is forwarded to the response-selection module of procedural WM, which carries out the updating operation. Thus, the first updating operation is carried out partially in parallel with the retrieval of the second item. This scheduling reflects the assumption that declarative WM and procedural WM are separate subsystems that can operate without mutual interference. Procedural WM does not have to wait until declarative WM finished retrieving both items.

The response-selection module has an input layer of 18 units (nine digits, nine locations), and an output layer of 18 units (nine digits, nine locations). On every updating step, the binding matrix connecting these two layers is configured to implement the updating operation to be carried out. For instance, an operation to add two to the retrieved digit is implemented by a matrix of bindings between every digit unit in the input layer and the unit of that digit plus two in the output layer. With that binding matrix in place, updating proceeds by activating the digit that has been retrieved in the item-selection module in the input layer of the response-selection module. From there, activation is fed through the binding matrix into the digit unit in the output layer that corresponds to the retrieved digit plus two. This activation is accumulated until the first unit in the output layer reaches the boundary. Apart from occasional distortion by noise, this is the unit representing the correct result of the updating operation. Once the first unit in the output layer of the response-selection module reached the boundary, the first updating step is completed.

As soon as the second item has been retrieved in the item-selection module, it too is forwarded to the response-selection module, and the second updating operation is initiated. I assume that updating of one digit and one spatial location can occur in parallel in procedural WM. Two parallel processes in the response-selection module might proceed at a slowed rate because of resource sharing (Tombu and Jolicoeur, 2003), but in the current simulations the rate of accumulation in the output layer of the response-selection module is not assumed to be resource-dependent. Parallel response selection means that the second updating operation does not have to wait until the first updating operation is completed; rather it starts as soon as the second item has been retrieved. The entire updating step is completed once both updating operations have been completed. The simulated response time for an updating step therefore is the time until the first digit accumulator *and* the first location accumulator in the response-selection module have reached the boundary.

The updating of declarative WM requires that the outcome of the updating operations, that is, the digit and the location selected in the response-selection module, are fed back to the item-selection module and bound to the current cues (i.e., the new digit must be bound to the currently activated musical-instrument unit, and the new location must be bound to the currently activated color unit). This process of updating the bindings in the item-selection module can be implemented by rapid delta-rule learning, which simultaneously adds the new bindings and removes the old bindings between the currently activated cues and the old digit and location. To keep the present simulations simple and comparable to the simulations of the dual-access paradigm (which does not involve updating), I did not implement this actual updating process. Rather, I simulated a version of the experiment in which the initial digits and dot locations remained unchanged in declarative WM, and the results of the “updating” operations are merely reported as an overt response, as in the dual-access paradigm.

The panels on the right side of Figure 7 show the simulation results for the four object-repetition conditions of Oberauer and Bialkova (2011), for updating steps with separate and with bound pairs. The model accurately reproduced the additive repetition benefits for separate pairs, and the over-additive pattern for bound pairs. The repetition benefits in this paradigm arise from two sources: Priming of the cues in the context layer, and strengthening of bindings between the retrieved items and the currently activated cues after each updating step. These two beneficial effects jointly over-compensate the effect of the inhibition of the retrieved items (i.e., the temporary reduction of their activation in the candidate layer). Therefore, there is a net benefit of repetition. The difference in repetition benefits between bound and unbound pairs reflects the different degrees of parallelism of item retrieval for those kinds of pairs. Completely parallel retrieval engenders the over-additive interaction that is evident for bound pairs. Even relatively modest deviations from complete parallelism change that pattern toward an additive one, seen for unbound pairs.

One prediction following from the model is that RTs are faster for bound pairs than unbound pairs because parallel retrieval is finished faster than serial or partially parallel retrieval. This prediction was borne out by the data, as can be seen in Figure 7. A further, more subtle prediction is that RTs on updating steps on unbound pairs are faster when they follow an updating step with a bound pair than when they follow a step with an unbound pair. This prediction arises because after updating a bound pair, the system only gradually slides back from parallel retrieval toward serial retrieval. This prediction cannot be directly tested in the data of Oberauer and Bialkova (2011) because the comparison is confounded with the object-repetition conditions: The transition from a bound to an unbound pair necessarily involves one repetition and one switch, whereas the transition from an unbound pair to an unbound pair involves either two repetitions or two switches. For an indirect test, I compared RTs on bound-unbound transitions to RTs on unbound-bound transitions. Both transitions involve one repetition and one switch. Mean RT on unbound pairs following bound pairs was 2.62 s (*SD* = 0.60), whereas RT on bound pairs following unbound pairs was 2.72 (*SD* = 0.57). Thus, updating of an unbound pair following a bound pair was not slower than updating of a bound pair. Assuming that the latter occurs in parallel, the former is likely to occur in a still largely parallel mode, reflecting a very gradual shift back into serial processing. Future experiments might test the prediction of a gradual slowing of RTs on unbound pairs after processing a bound pair more directly.

One could ask why participants in the arithmetic dual-access paradigm always retrieve the two digits in parallel, whereas in the present dual-operation condition they tend toward serial retrieval except when a bound pair is presented. At present I can only offer some speculations on this question. One important difference between the two cases is that in the dual-access paradigm, the two retrieved items are combined as input for a single cognitive operation, whereas in the dual-operation condition they are treated separately as inputs to two independent updating operations. When the WM system needs to carry out two independent processing streams of item retrieval followed by response selection, letting those streams run in parallel incurs the risk of cross-talk: The retrieved items could be blended or confused, so that they influence each other's response-selection process. In the updating paradigm of Oberauer and Bialkova (2011) the risk of crosstalk is minimal because the two items are maximally different from each other—a confusion between a digit and a spatial location is highly unlikely. Nevertheless, the WM system might have a strong default preference for scheduling two independent process streams serially, or at best semi-parallel, to reduce their temporal overlap and thereby the risk of cross-talk. This default preference can be overcome with extensive practice (Oberauer and Kliegl, 2004), but apparently only for bound pairs (Oberauer and Bialkova, 2011).

Another, related reason for the strong tendency of the WM system to temporally separate two process streams is that each process stream involves the (re-)configuration of the response-selection module to implement the appropriate updating operation. Two such reconfiguration processes have to be carried out on every updating step, one to implement the appropriate arithmetic operation (i.e., adding 2 when the tone is high, and subtracting one when the tone is low), and one to implement the required spatial updating operation (e.g., shift the retrieved dot location to the left). For the present simulations I did not model these task-set reconfiguration processes explicitly—I simply assumed that the appropriate task sets are in place in the response-selection module—but it is at least plausible that the cognitive system has a bias in favor of scheduling cognitive operations sequentially when they require two separate reconfiguration processes in procedural WM (Meyer and Kieras, 1997; Oberauer and Bialkova, 2011). In contrast, in the dual-access paradigm a single operation needs to be carried out in procedural WM, requiring only a single reconfiguration process (i.e., switching between addition and subtraction). Thus, there is no risk of cross-talk in procedural WM. To the contrary, the arithmetic operation in procedural WM requires activation of a chunk, and that chunk can only be activated by two items providing input simultaneously. If the activation level of an item retrieved in the item-selection module cannot be sustained indefinitely, parallel retrieval would be advantageous for synchronizing the activation of the two digits that are required to jointly activate the corresponding chunk unit.

### Dual-access again, with parallel and serial access

**Figure 9:**
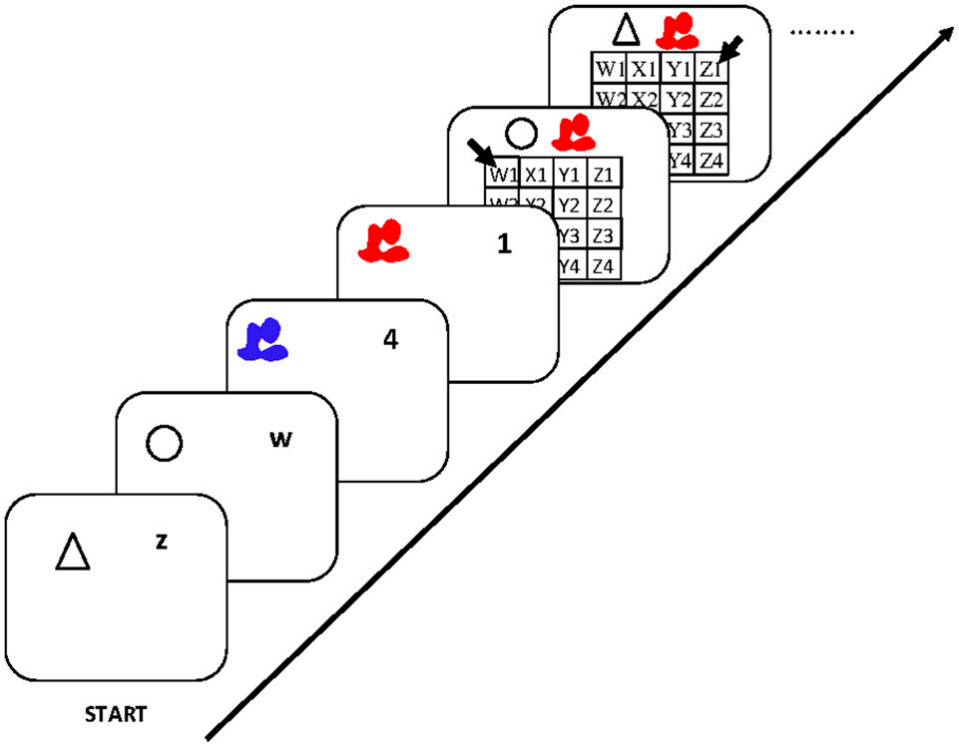
**Flow of events in the experiment of Gilchrist and Cowan (2011).** Participants initially encoded two associations between a shape and a letter (w, x, y, z), and two associations between a color and a digit (1, 2, 3, 4). They then were presented a pair of a shape and a color, and had to retrieve the associated letter and digit, respectively. They responded by selecting the correct letter-digit pair by mouse click in a matrix.

My final simulation applies the model to a paradigm that combines features of the arithmetic dual-access paradigm and the dual-operation updating paradigm discussed above. Gilchrist and Cowan (2011) published a series of experiments with a modified dual-access paradigm (see Figure 9) that demonstrated largely additive repetition benefits, in contrast to the findings of Oberauer and Bialkova (2009). Gilchrist and Cowan asked participants to remember four pairs of stimuli. Two pairs were conjunctions of a geometric shape (triangle or square) with a letter (w, x, y, or z) and the other two were conjunctions of a color patch (red or blue) with a digit (1, 2, 3, or 4). After encoding these four conjunctions, participants worked through a series of trials, on each of which they saw one shape together with one color patch. They had to make a speeded response by clicking with the mouse into the appropriate cell of a 4 × 4 grid. Each cell was labeled by a pair of letter-number coordinates (i.e., w1, w2, …, z3, z4). Participants had to retrieve the letter associated to the given shape and the digit associated to the given color, and thus determine the correct response cell. There were four conditions of transitions from one trial to the next, generated by crossing repetition or switch of the letter with repetition or switch of the digit. Gilchrist and Cowan found repetition benefits when both the letter and the digit were repeated, consistent with our results (Oberauer and Bialkova, 2009), but they also found (smaller, but significant) repetition benefits when only the letter, or only the digit, was repeated, contrary to our findings.

**Figure 10:**
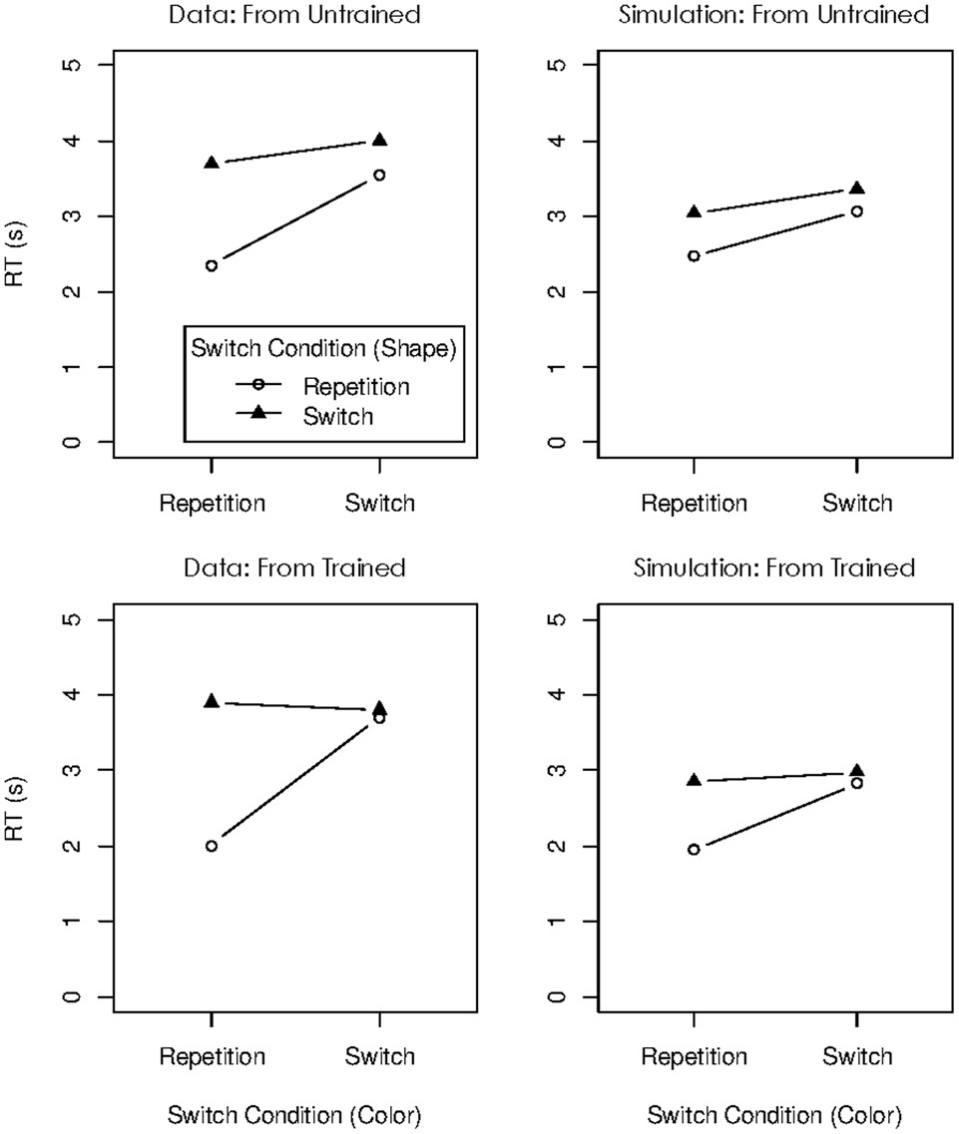
**Experimental data (left) and simulated data (right) for Experiment 3 of Gilchrist and Cowan (2011)**.

In their Experiment 3, Gilchrist and Cowan (2011) trained participants on two color-shape combinations (e.g., a red circle), each of which was consistently associated with one letter-digit combination (e.g., Y4). Gilchrist and Cowan assumed that this procedure enabled the formation of a chunk of each trained color-shape combination, together with the associated letter-digit combination. In the test phase of the experiment, the stimuli for each trial (i.e., the color-shape combinations cueing the response) could either be a trained combination or an untrained combination. For those trials that immediately followed after trials using a trained stimulus combination, Gilchrist and Cowan observed large repetition benefits when both elements were repeated (e.g., a red circle followed by a red circle), but no benefit when only one element was repeated (e.g., a red circle followed by a red square), fully consistent with the pattern in our study with arithmetic dual-access (Oberauer and Bialkova, 2009). In contrast, for trials following trials using an untrained stimulus combination, Gilchrist and Cowan again found a benefit for repetition of a single element (see Figure 10). They concluded that chunking undermines the repetition benefit in transitions where only one element is repeated, because when trial *n*-1 used a chunk rather than separate representations of each element, then there can be no advantage of re-using a representation of the repeated element on trial *n*. In contrast, when not-chunked elements are used in trial *n*-1, repeating any one of them yields a benefit because processes in trial *n* can build on the representation of an element already in the focus of attention. According to Gilchrist and Cowan, this result shows that two, and possibly more, items can be held in the focus of attention simultaneously.

I don't believe that the brief training episode in Experiment 3 of Gilchrist and Cowan (2011)—a mere 10 repetitions of each color-shape combination—is sufficient for the formation of a chunk comparable to the two-digit chunks acquired from a lifetime of practice with basic arithmetic. In confirmation, RTs from trials using a trained stimulus combination were not significantly faster than those from trials using untrained stimulus combinations, whereas chunking should lead to a considerable speed advantage for trained stimulus combinations. I think that the practiced combinations in that experiment are more comparable to the “bound” representations in previous studies (Bao et al., 2007; Oberauer and Bialkova, 2011). Therefore, my simulation of the Gilchrist-Cowan version of the dual-access paradigm builds directly on the simulation of the dual-operation condition of Oberauer and Bialkova (2011), using the same rule for adjusting cue weights (Equation 1) with the same parameter values, *C*_min_ = 0.4, and *C*_r_ = 0.8. The item-selection module consists of a context layer with four units (two colors, two shapes), and an item layer with eight units (four letters, four digits), each linked to a corresponding unit in the candidate layer.

At the beginning of each trial, the units for the presented color and the presented shape in the context layer are activated according to the current cue weights. Item retrieval proceeds more or less in parallel, depending on the cue weights. Once the first item is retrieved, it is immediately forwarded to the response-selection module, where it activates the corresponding unit in the input layer. The input layer of the response-selection module consists of the same eight units as the item layer (four letters, four digits). Response selection in the Gilchrist-Cowan paradigm can be thought of as a visual search process: Given the retrieval of a letter, the response-selection module searches for the row or column in the response grid that contains that letter, and given a retrieved digit, it searches for the column or row that contains that digit. I modeled the task set for these two visual-search processes in a highly simplified way: Each retrieved letter feeds activation into one of four accumulators in the output layer, each of which stands for the column or row in which one letter is currently displayed. Likewise, each retrieved digit feeds activation into one of another set of four output units, representing the row or column containing that digit. The two response-selection processes can run in parallel as in the preceding simulation, and the response is given once they have both finished, implying that the location of both the letter and the digit has been found in the grid.

The cue weights are adjusted from trial to trial according to Equation 1, using the same parameter values. That is, when a trained color-shape combination is shown, the cue weights are set to [1, 1], implementing fully parallel retrieval of the letter and the digit. When a not-trained color-shape combination is shown, the weaker cue weight is reduced proportionally, so that retrieval gradually slides toward a more serial schedule. As a consequence, on trials immediately following a trial with a trained (and thus bound) combination, the cue weights are still set to largely parallel retrieval. On trials immediately following a trial with an untrained (and thus unbound) combination, cue weights are set, on average, to values implying more serial retrieval: The majority of those trials involve again a not-trained combination, and hence, the cue weights slide more toward the serial setting. As a consequence, the repetition benefits show a pattern closer to over-additive repetition effects on trials that follow upon trained trials, and a more additive pattern on trials that follow upon not-trained trials. This is the pattern observed by Gilchrist and Cowan (2011), reproduced in the left panel of Figure 10. It is reproduced by the simulation of their Experiment 3, displayed in the right panels of Figure 10.

Whereas Gilchrist and Cowan reported that on trials following trained trials, the repetition benefit for repeating a single element disappeared, in the simulations this benefit is still present, though small. This discrepancy might be only apparent, because the lack of a statistically significant repetition benefit in Experiment 3 of Gilchrist and Cowan does not imply that the repetition benefit is entirely abolished—it might just be a small effect that is easily obscured by noise in a single experiment.

## Discussion

The goal of this article was to apply a computational model of declarative and procedural WM (Oberauer et al., 2013) to situations where people need to access two items in WM to complete a task. I have simulated RT data from three paradigms that require access to two items in declarative WM. Different patterns of RTs emerging from these three paradigms had previously been used to argue for contradictory conclusions about how many items can be held in the focus of attention simultaneously. The model proved to be a useful common framework for explaining the diverse and potentially confusing set of results.

I am far from confident that the individual models I developed for the three paradigms are adequate for these paradigms. The present simulations show that these models are sufficient to explain the currently available data from the three paradigms, but so far we have far too little data to thoroughly evaluate the models. The successful application of the general model architecture to a new set of findings from three experiments demonstrates that the model architecture provides a useful framework for approaching complex WM tasks through computational modeling. Within this architecture, I tried dozens of model variants for the three paradigms, and the ones I presented here were the only ones that gave an acceptable account of the experimental data without making a large number of arbitrary additional assumptions. Therefore, I suspect that some key features of the present models—in particular the assumptions about serial or parallel retrieval—are not only sufficient but also necessary to explain the data, although there is no way to prove this necessity.

The starting point of the present endeavor was the question: How many items can be held in the focus of attention in WM simultaneously? Readers will rightly expect an answer to this question. The answer will not be a number, though. We first need to identify the focus of attention in the computational model. My suggestion is to regard the representations activated in the item layer and the context layer of declarative WM at any moment in time as the current content of the focus of attention. During processing of a task, the activation level of representations in these two layers fluctuates. In the context layer, one or several representations can become activated by perceptual input; depending on the cue weights, these contexts are activated to different degrees. In addition, residual activation from the preceding trials remains in the context layer. Hence, the focus of attention can contain several context cues at the same time, with varying degrees of activation. Activation in the item layer is more restricted because the item layer is entirely reset to zero after each processing step. Yet, retrieval of an item involves a race of activation accumulation of all units in the item layer, so all of them are activated to some extent, if only by noise. Moreover, when two items are retrieved in parallel, or semi-parallel, as in the simulations presented here, then two items become fairly strongly activated at about the same time during each retrieval step. Hence, the focus of attention can also contain several active item representations at the same time, again with varying degrees of activation. There is no structural limitation to the contents of the focus of attention in the model architecture.

Any limitation of the focus of attention to a single context and/or a single item arises from the function of the focus as a selection device. The focus of attention serves to represent the context and content (or item) representations that are needed at any moment in time. When the task requires selecting one item cued by one context, then only one context representation will be highly activated in the context layer, resulting in one item becoming highly activated in the item layer—unless the cue is not distinctive enough and strongly activates more than one item. In that case, selection of the appropriate item is likely to fail. In this case, strongly activating more than one item is dysfunctional. In contrast, when the task requires access to two items, as in the paradigms investigated here, it can be functional to activate two context representations at the same time, resulting in simultaneous strong activation of two item representations. Whether or not such a parallel or semi-parallel processing schedule is feasible depends on the risk of cross-talk between the representations that are activated at the same time. The risk of cross-talk is minimal when one of two conditions is met: One is that the two items to be retrieved are jointly needed for processing, and there is no need to assign each of them a specific role, so that confusing them has no detrimental consequences. This condition is met in the arithmetic dual-access paradigm: Both digits are needed to activate the corresponding chunk, and the two digits play exchangeable roles in doing so. If the task were only slightly changed—for instance, requiring people to report the signed difference rather than the absolute difference of two digits—then confusion between the two retrieved digits would wreak havoc. The WM system would thereby be forced to shift to a more serial processing schedule. The second condition under which cross-talk is minimized is when two highly distinct items must be retrieved based on two highly distinct cues. This condition is met in the paradigms of Oberauer and Bialkova (2011) and of Gilchrist and Cowan (2011).

One prediction following from these considerations is that whenever the two conditions above are not met, the WM system relies on a largely serial processing schedule, accessing and processing items one by one. As a consequence, the RT benefits for retrieval or processing of each item should be additive. Such benefits can arise from item (and context) repetition, as in the paradigms discussed here, but they can also arise from other experimental manipulations such as the degree of learning of individual item-context bindings, or the difficulty of operations applied to individual items.

Whether or not two (or more) items and their contexts are highly activated simultaneously in the item-selection module, the maintenance function of WM is not served by this activation but rather by the bindings between items and contexts in the binding matrix. The binding matrix of the item-selection module is the computational implementation of the region of direct access in the model. Whether or not an item in a memory set can be retrieved depends on the relative strength of its binding to the context that is used as a retrieval cue to that item.

Looking back at the theoretical starting point of the present investigation, we can now clearly see how the container metaphor for the focus of attention is misleading. Based on the container metaphor, Svetlana Bialkova and I took the over-additive interaction of item-repetition benefits as evidence for a focus of attention that holds only one chunk at a time (Oberauer and Bialkova, 2009). In light of the present modeling work, the intuition that chunking plays a role in this pattern is correct, but the conclusion is wrong: the over-additive pattern is indicative of parallel retrieval of two digits, implying that two items are simultaneously highly active in the item layer of declarative WM. These items are not chunked in declarative WM, they are two separate representations, and hence, two separate items are “in” the focus of attention. The chunk representation of digit pairs in procedural WM plays an additional role in strengthening the over-additive pattern of repetition benefits but it is not necessary for generating it.

The reverse argument holds for Gilchrist and Cowan (2011). Based on their finding of repetition benefits for repetitions of just one out of two stimuli, they concluded that two items can be held in the focus of attention simultaneously. The present model-based analysis revealed that this empirical pattern is indicative of serial or semi-parallel retrieval. Hence, the RT pattern of Gilchrist and Cowan does not imply that two items are highly active in the item-layer at the same time. The independent repetition benefits for repeating each element arise from the residual activation of the two cues (i.e., the shape and the color) from the previous trial in the context layer, and the strengthened cue-item bindings of these cues. Thus, one could conclude from the results of Gilchrist and Cowan that two context cues from the preceding trial remain partly activated in the context layer, carrying over into the current trial. I don't think, however, that this state of affairs is adequately characterized by saying that two items are held in the focus of attention.

To conclude, in light of the present modeling results the two RT patterns from the dual-access experiments reported by Oberauer and Bialkova (2009) and by Gilchrist and Cowan (2011) imply, if anything, the opposite of what the authors originally inferred from them. The strong interaction pattern of Oberauer and Bialkova indicates that more than one item is retrieved in parallel, and thus could be said to be in the focus of attention, whereas a more additive pattern as obtained by Gilchrist and Cowan indicates less temporal overlap of item retrieval.

More importantly, proceeding from metaphors to mechanisms changes the way we frame the problems to be addressed. To be or not to be in the focus of attention no longer is the question of foremost interest. Rather, we should ask how sets of items are represented in WM and how individual items can be accessed from such a representation in an efficient way. Approaching the representational states in WM through computational modeling is likely to deepen our understanding not only of object-repetition effects but also of other empirical phenomena attributed to the focus of attention in WM, such as people's limited capacity for short-term maintenance (Cowan, 2011), the increased retrieval rate for the last-presented item in short-term recognition (McElree, 2006), and the facilitation of access to WM representations by a retro-cue given in the retention interval (Lepsien et al., 2011; Rerko and Oberauer, 2013).

## Author note

The Matlab code running the simulations reported in this article is available from the author's web page: www.psychologie.uzh.ch/fachrichtungen/allgpsy/Team/Oberauer_en.html

### Conflict of interest statement

The author declares that the research was conducted in the absence of any commercial or financial relationships that could be construed as a potential conflict of interest.

## Footnotes

^1^The two-digit chunks assumed to underlie mental arithmetic differ from the chunks representing memory sets or task sets in the set-selection module of my model: Arithmetic chunks are implemented by localist representations in the response-selection module, whereas set chunks are distributed representations in the set-selection module. Future modeling work will have to show whether the two kinds of chunks can be related to each other more systematically.

^2^Participants in the arithmetic dual-access experiments knew that the result was always between 1 and 9, so computing the absolute difference was sufficient. If participants had to compute the signed difference rather than the absolute difference, they could not have used unordered chunk representations for the subtraction task, and there would have been no chunk priming between addition and subtraction tasks using the same digits.

^3^The expected value of the maximum of two random variables becomes smaller when the mean of one of them is reduced, and the amount of reduction in the expected value of the maximum increases as the variance of the two variables increases. The reduction is always smaller than the reduction in the mean of the single variable.
